# Deciphering the complete human-monkeypox virus interactome: Identifying immune responses and potential drug targets

**DOI:** 10.3389/fimmu.2023.1116988

**Published:** 2023-03-27

**Authors:** Raghav Kataria, Simardeep Kaur, Rakesh Kaundal

**Affiliations:** ^1^ Department of Plants, Soils, and Climate, College of Agriculture and Applied Sciences, Logan, United States; ^2^ Bioinformatics Facility, Center for Integrated BioSystems, Logan, United States; ^3^ Department of Computer Science, College of Science, Utah State University, Logan, UT, United States; ^4^ Division of Biochemistry, Indian Agricultural Research Institute (ICAR), New Delhi, India

**Keywords:** monkeypox virus, protein-protein interactions, computational modelling, drug targets, immune response, human

## Abstract

Monkeypox virus (MPXV) is a dsDNA virus, belonging to Poxviridae family. The outbreak of monkeypox disease in humans is critical in European and Western countries, owing to its origin in African regions. The highest number of cases of the disease were found in the United States, followed by Spain and Brazil. Understanding the complete infection mechanism of diverse MPXV strains and their interaction with humans is important for therapeutic drug development, and to avoid any future epidemics. Using computational systems biology, we deciphered the genome-wide protein-protein interactions (PPIs) between 22 MPXV strains and human proteome. Based on phylogenomics and disease severity, 3 different strains of MPXV: Zaire-96-I-16, MPXV-UK_P2, and MPXV_USA_2022_MA001 were selected for comparative functional analysis of the proteins involved in the interactions. On an average, we predicted around 92,880 non-redundant PPIs between human and MPXV proteomes, involving 8014 host and 116 pathogen proteins from the 3 strains. The gene ontology (GO) enrichment analysis revealed 10,624 common GO terms in which the host proteins of 3 strains were highly enriched. These include significant GO terms such as platelet activation (GO:0030168), GABA-A receptor complex (GO:1902711), and metalloendopeptidase activity (GO:0004222). The host proteins were also significantly enriched in calcium signaling pathway (hsa04020), MAPK signaling pathway (hsa04010), and inflammatory mediator regulation of TRP channels (hsa04750). These significantly enriched GO terms and KEGG pathways are known to be implicated in immunomodulatory and therapeutic role in humans during viral infection. The protein hubs analysis revealed that most of the MPXV proteins form hubs with the protein kinases and AGC kinase C-terminal domains. Furthermore, subcellular localization revealed that most of the human proteins were localized in cytoplasm (29.22%) and nucleus (26.79%). A few drugs including Fostamatinib, Tamoxifen and others were identified as potential drug candidates against the monkeypox virus disease. This study reports the genome-scale PPIs elucidation in human-monkeypox virus pathosystem, thus facilitating the research community with functional insights into the monkeypox disease infection mechanism and augment the drug development.

## 1 Introduction

Monkeypox, a rare viral disease first reported in a 9-month-old baby boy from the Democratic Republic of Congo in 1970, started causing an epidemic fear in the early and mid 2022, immediately as the COVID-19 pandemic subsided (1). Previously endemic to the African regions, the current severe outbreak of monkeypox is prevalent in countries near Europe and Western hemisphere (Kaler et al., 2022; Reed et al., (2)). Monkeypox virus, a Poxviridae family virus belonging to the genus Orthopoxvirus, is a dsDNA virus with slight pleomorphic characteristics and possess a dumbbell-shaped core with lateral bodies (3). According to current reports, these viruses have two major genetic clades: Central African or Congo Basin and the Western African, with the former being more virulent and causing more disease severity (4). Although the current spread of MPXV for the general public is very low, studies are currently underway to further understand the epidemiology, sources of infection, and transmission patterns.

MPXV can be transmitted in several ways, all of which involve direct contact with the infected organism (human or animal) (3). Animal to human transmission of monkeypox can be considered as primary mode of transmission, which can be due to the direct exposure to infected animals *via* bites or scratches, cooking and consumption of infected animals, and any contact with cutaneous or mucosal lesions (5). Although human to human (secondary transmission) mode of transmission is less common than primary transmission, it primarily involves respiratory droplets in close contact or direct contact with cutaneous lesions of an infected person. Recently, the contaminated surfaces are also thought to be major risk factors for monkeypox transmission among humans (6). According to the World Health Organization (WHO), transmission can be attributed to close contact with infected individuals; however, the sexual mode of transmission of this disease is unknown.

**Figure 1:**
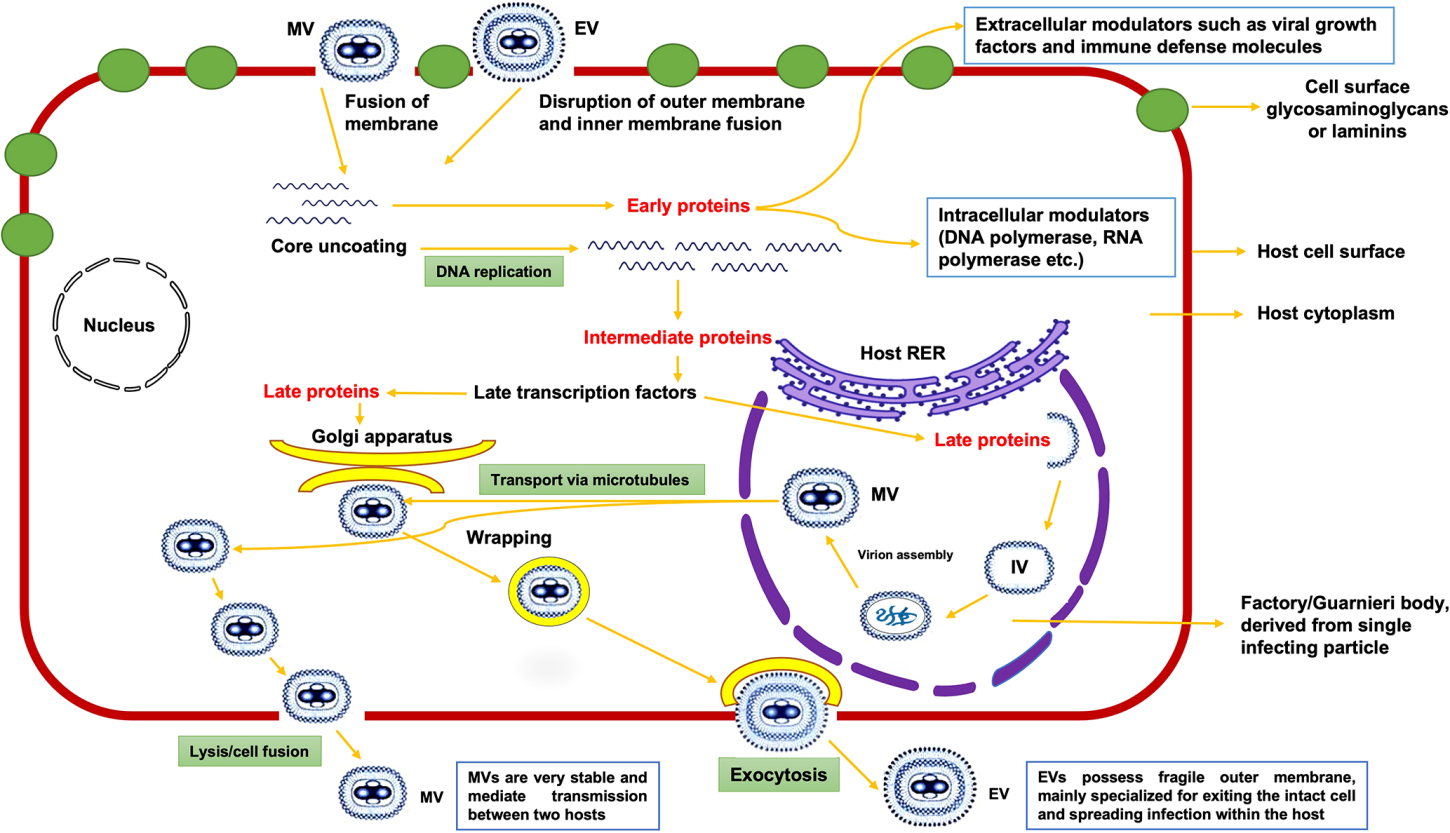
Proposed model of replication of monkeypox virus inside the host cytoplasm; MV, IV, and EV denotes Mature Virion, Immature Virion, and Enveloped Virion, respectively.

Unlike other viruses, MPXV replication occurs in the cytoplasmic structures known as factories/Guarnieri bodies of the host cell (7). Both mature virion (MV) with a single membrane and enveloped virion (EV) with an outer membrane have proteins that aid in binding to a host cell *via* membrane fusion and entry into the cytoplasm of host (8). Viral proteins from MV facilitate its attachment to host cell membrane by binding to laminin or glycosaminoglycans present on the membrane (Moss, (9)). MPXV replicates inside the host cell in factories, where each factory (compact structure of DNA) is derived from one infecting particle and its membrane is derived from the rough endoplasmic reticulum (RER) of host in early-stage infections (10). During the replication process, these factories enlarge and become irregular in shape, with small cavities containing virus mRNA and host translation factors, eventually forming immature virion (IV) assembly (7, 11). Lastly, IV is converted into MV (the most infectious virion), which exits the host cell *via* lysis/cell fusion (**Figure 1**), and is primarily responsible for mediating the disease transmission between host species. Enveloped virions, on the other hand, have a fragile membrane on their surface and are mainly specialized for spreading infection within the host (12).

**Figure 2:**
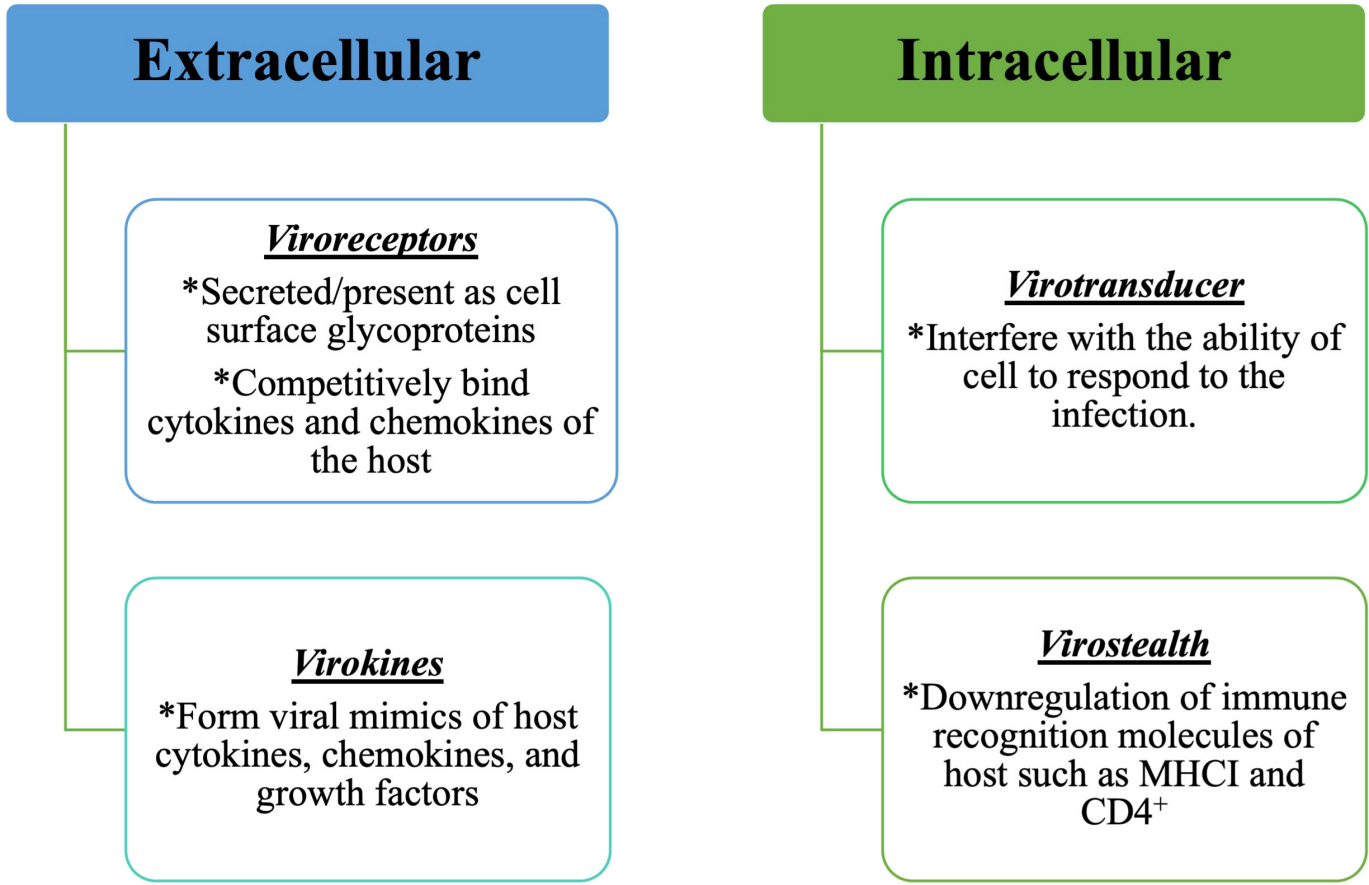
Monkeypox proteins responsible for modulatory action against immune response of the host.

The molecular interactions between host and pathogen cells serve a variety of functions, including immune response to several infectious diseases. Pathogens secrete effector molecules into the host cell, subvert the intercellular mechanisms of the host cell and cause the infection (13). MPXV employs a set of modulatory proteins encoded by its virulence genes to evade the immune system of the host (1). Based on functioning of the modulatory proteins, these can be classified into two groups — intracellular and extracellular. Intracellular proteins include both virotransducers that work by interfering with the ability of cell to respond to infection, and virostealth proteins, which decrease the likelihood of the virus being detected by the host immune system by downregulating immune recognition molecules such as MHC class I and CD4^+^ (14). Extracellular proteins include viromimic proteins that function to modulate the immune system response. **Figure 2** describes classification and functions of two types of modulatory proteins. These modulatory proteins work in tandem to allow viral replication while evading the host immune system, but in the absence of these proteins, virus will be unable to evade the immune system.

Protein-protein interactions between host and pathogen play a crucial role in the understanding of infection mechanism and the subsequent host cell immune response. Therefore, to gain deeper insights into the disease infection mechanism of MPXV in humans, we aim to develop computational models to decipher genome-scale protein-protein interactions in human-monkeypox virus pathosystem. These models include homology-based interolog and domain-based prediction, which have been widely used in several host-pathogen interaction studies in the past (15, 16). The molecular techniques available for the detection and/or validation of PPIs are laborious and costly, while the computational approaches, on the other hand, provide a comprehensive understanding of the biological function and cellular behaviour of the proteins involved in the interactions in rapid and economical manner (17). To the best of our knowledge, our study is the foremost report that provides novel insights into the sequence-based PPIs prediction between human and various monkeypox virus strains using computational approaches, and unravelling the candidate drug targets in human against MPXV.

## 2 Materials and methods

### 2.1 Dataset collection

To study the protein-protein interactions, the human proteins were analyzed against multiple strains of monkeypox virus. The human protein data was obtained from UniProt (Swiss-Prot) (https://www.uniprot.org/), while the proteomes of MPXV strains were obtained from NCBI (https://www.ncbi.nlm.nih.gov/). For MPXV, we obtained the proteomes of all the strains that caused MPXV outbreak in 2017-2018 (https://ncbiinsights.ncbi.nlm.nih.gov/2022/05/26/monkeypox-virus-genome/). Additionally, we also considered the strains from the countries with number of cases more than 100 (https://www.cdc.gov/poxvirus/monkeypox/response/2022/world-map.html) as of August 23, 2022. Combining information from both the sources above, 22 MPXV strains were considered for further analysis. All the proteomes were analyzed with CD-HIT (Cluster Database at High Identity with Tolerance) (18) at 100% identity to avoid redundancy. The detailed information about the protein datasets of MPXV strains is available in **Table 1**.

**Table 1:** Detailed information of the monkeypox virus strains studied.

Year	Country	Monkeypox virus Strain/Isolate	NCBI/GenBank accession	Number of proteins	
				Downloaded	CD-HIT (100%)
2006	Democratic Republic of the Congo	DRC 06-0999	JX878409.1	191	187
2007	Democratic Republic of the Congo	DRC 07-0514	JX878428.1	191	187
2007	Democratic Republic of the Congo	DRC 07-0354	JX878425.1	190	186
2017	Central African Republic	A5_contig_SPADES	MN702444.1	170	170
1996	Democratic Republic of the Congo	Zaire-96-I-16	NC_003310.1	191	187
2003	USA	USA_2003_039	DQ011157.1	198	191
1978	Nigeria	W-Nigeria	KJ642615.1	176	172
2018	Nigeria	MPXV-M5312_HM12_Rivers	MT903340.1	181	177
2018	UK	MPXV-UK_P2	MT903344.1	181	177
2019	Singapore	MPXV-Singapore	MT903342.1	181	177
2018	UK	MPXV-UK_P1	MT903343.1	181	177
2018	UK	MPXV-UK_P3	MT903345.1	181	177
2022	USA	MPXV_USA_2022_MA001	ON563414.3	190	186
2022	Austria	hMPXV/Austria/MUW1525179/2022	OP019275.1	179	175
2022	Canada	MPX/2022/Canada/AB1	ON736420.2	214	209
2022	Germany	MPXV/Germany/2022/RKI249	OP215228.1	179	175
2022	Ireland	WA-2022	ON872184.1	143	142
2018	Israel	Israel_2018	MN648051.1	213	208
2022	Italy	INMI-Pt2	ON745215.1	190	186
2022	Netherlands	hMpxV/Netherlands/NH-AUMC-0001/2022	OP160532.1	179	175
2022	Portugal	MPX/PT0049/2022	OP245306.1	175	171
2022	UK	MPXV_UK_2022_5	OP022170.1	179	175

### 2.2 Computational models for interactome prediction

The advancement in the bioinformatics techniques allows us to analyze big data with high efficiency. In this study, the host-pathogen interaction prediction was performed by implementing two protein sequence-based approaches; interolog and domain-based. The homology-based “interolog” approach relies on the sequence similarity of the proteins. Using BLASTp v2.7.1, the human and MPXV proteomes were aligned against the gold standard PPI databases (BioGRID, DIP, HPIDB, IntAct, MINT, and VirHostNet) that contain protein interaction information. To improve the accuracy of the PPI prediction, these databases were filtered for human-virus interactions only. Following the BLAST search, we generated 100 random combinations using the BLAST parameters: sequence identity (40%, 50%, 60%, 70%, 80%), *e*-value (1*e*-03, 1*e*-05, 1*e*-10, 1*e*-20), and sequence coverage (40%, 50%, 60%, 70%, 80%) to filter the BLAST results. The filtered BLAST results were then employed for PPI prediction using in-house python scripts and local SQL databases.

On the other hand, the “domain-based” approach is based on the protein domains, which are employed to infer the interactions between host and pathogen (19). The significant domains were extracted from the host and pathogen proteins using HMMER v3.3.2 against Pfam database (pfam35). The extracted domains were filtered with an *e*-value of 1*e*-23 and coverage of 0.2 for human, and an *e*-value of 1*e*-01 and default coverage for virus proteins. These domains were then queried against the standard domain-domain interaction (DDI) databases (3did, DOMINE, and IDDI) for interactions prediction. The number of interactions in the gold-standard databases have been summarized in **Supplementary Material 1**, **Sheet 1**.

### 2.3 Functional analysis

Gene ontology (GO) and Kyoto Encyclopedia of Genes and Genomes (KEGG) enrichment provide deeper insights into the molecular functions of the proteins and their respective biological pathways. GO and KEGG enrichment of the proteins in the predicted interactions was performed using the R package, ClusterProfiler (20), with an adjusted *p*-value cutoff of ≤0.05, and the subsequent plots were generated using R package “ggplot2”. The proteins synthesized by the cellular machinery are translocated to various compartments in the cell, whereby each protein performs a specific function, thus revealing the relationship between protein function and subcellular localization. In line with this, the subcellular localizations of the human proteins were obtained from Human Protein Atlas (https://www.proteinatlas.org/) (21), and UniProt (https://www.uniprot.org/).

### 2.4 Network visualization of proteins

The study of molecular interactions is critical to understand the interconnected subcellular mechanisms and molecular pathways that maintains cellular homeostasis (22). The analysis of the PPI network provides a holistic view of the biological and cellular processes. The protein hubs are immensely important in a network, owing to their high connectivity with the neighboring proteins (23). The global PPI network of human-MPXV interactome was visualized and the significant protein hubs were further analyzed using the most widely used tool— Cytoscape (24).

## 3 .Results and discussion

### 3.1 Phylogenetic patterns of different MPXV strains

**Figure 3:**
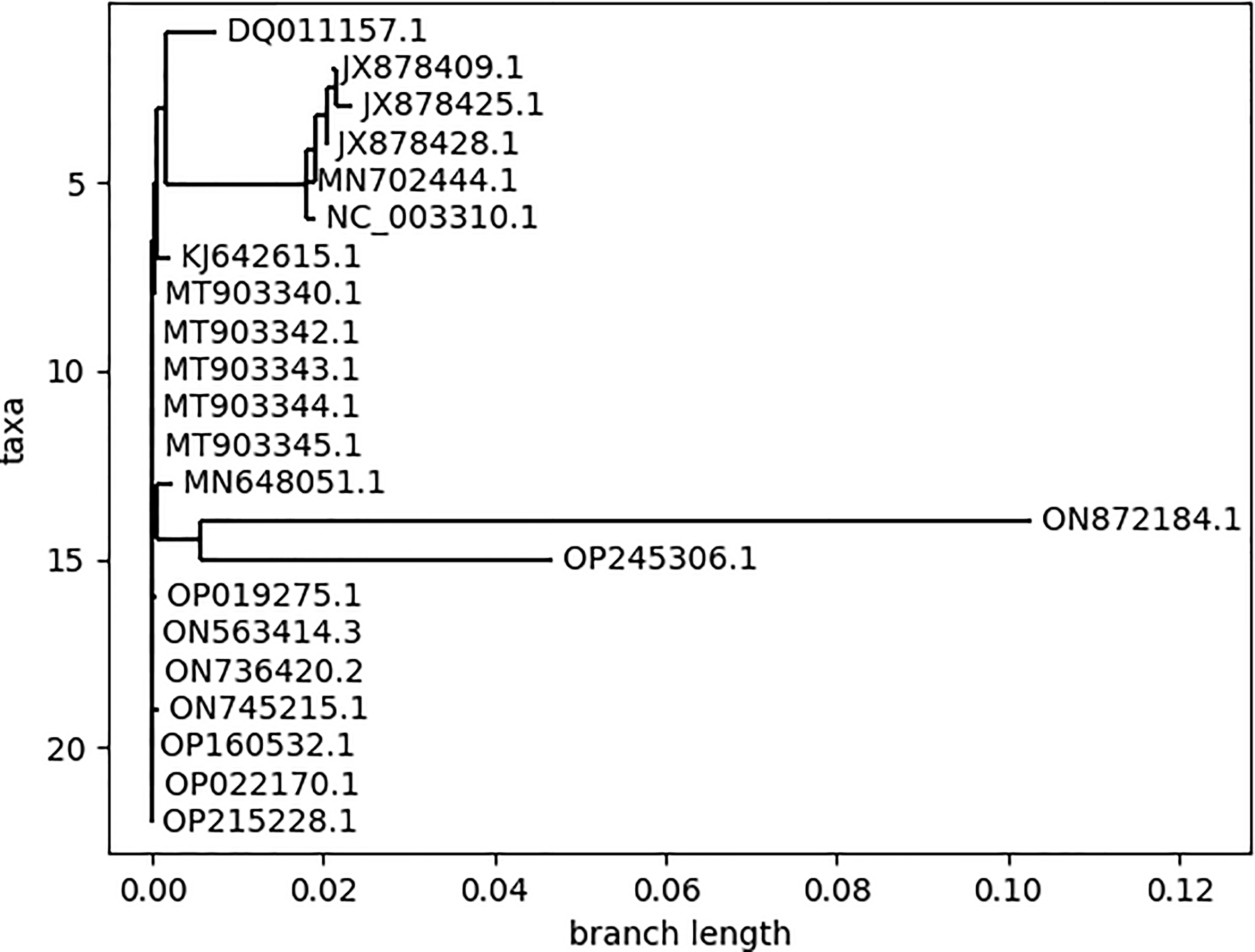
Phylogenetic tree of 22 monkeypox virus strains constructed using the respective genomes.

The phylogenetic classification reveals the evolutionary patterns between different species (25). The phylogenetic tree (**Figure 3**) was generated for all the MPXV strains for the selection of strains to be analyzed for further analysis. The pattern of branching in the phylogenetic classification resulted in three major clades and revealed insignificant differences between the strains within the individual clade. Based on this, we considered one representative strain from each clade, resulting in 3 strains belonging to different countries: Zaire-96-I-16 (Democratic Republic of the Congo; 1996), MPXV-UK_P2 (UK; 2018), and MPXV_USA_2022_MA001 (USA; 2022). Throughout the research analysis, we referred to these strains using their GenBank accessions: NC_003310 for Zaire-96-I-16, MT903344 for MPXV-UK_P2, and ON563414 for MPXV_USA_2022_MA001. Additionally, the strains ON872184.1 and OP245306.1 showed significant divergence as compared to other strains but due to the unavailability of the complete genome, these two strains were not considered for the downstream analysis.

### 3.2 Human-MPXV interactome: Computational prediction of PPIs

The implementation of the computational approaches for PPI prediction resulted in a significant number of potential PPIs between the proteomes of human and 3 strains of MPXV. The PPIs predicted from both the interolog- and domain-based models were combined, followed by the removal of the duplicate interactions to reduce the false positive interactions. To predict the interactome, we used the BLAST parameters combination with sequence identity 50%, *e*-value 1*e*-05, and sequence coverage 50%. These parameters are also implemented as default filtering options in HPIDB database to filter the BLAST results for PPI prediction (26). In the past, no standard cutoff has been established to filter the BLAST parameters for the prediction of highly confidential PPIs. The predicted interactome of human against each of the three strains is provided in **Table 2**. Furthermore, for comparative analysis, we extracted the common host proteins and performed the functional analysis separately for the common and unique host proteins obtained from the three strains.

**Table 2:** Protein-protein interactions prediction from Interolog- and Domain-based approaches using BLAST parameters: 50% sequence identity, 1*e*-05 *e*-value, and 50% sequence coverage.

Prediction method	Standard database	NC_003310			MT903344			ON563414		
		Interactions	Host	Pathogen	Interactions	Host	Pathogen	Interactions	Host	Pathogen
**Interolog**	**BioGRID**	21	20	4	20	19	3	21	20	4
	**DIP**	31	23	11	23	20	9	26	23	10
	**HPIDB**	1,144	940	48	1,122	932	42	1,129	936	44
	**IntAct**	372	269	23	366	269	22	366	269	22
	**MINT**	73	64	13	72	63	12	72	63	12
	**VirHostNET**	817	720	43	803	715	38	807	719	39
	**Interolog (Total)**	1,197	981	52	1,174	976	46	1,182	981	48
**Domain**	**3did**	8,644	3,165	76	5,994	3,165	68	6,196	3,264	74
	**DOMINE**	25,669	3,044	73	21,217	2,967	65	22,533	3,021	71
	**IDDI**	89,813	7,264	98	79,453	7,236	89	85,333	7,349	95
	**Domain (Total)**	97,696	7,441	106	85,452	7,416	96	92,154	7,522	102
**Interolog & Domain (combined)**		98,827	7,998	121	86,555	7,968	110	93,265	8,076	117

### 3.3 Protein kinases constitute major human protein hubs

The protein interaction networks are usually scale-free and contain various protein hubs that maintain the global structure of the PPI network. In a host-pathogen PPI network, the host/pathogen proteins are nodes and the interactions between the host and pathogen proteins are edges (27). The highly connected hubs are considered to be more biologically essential and play a role in maintaining the overall integrity of the network (28). The deletion of a significant node can result in the loss of a number of edges that might be crucial for understanding the molecular interaction environment. Thus, it shows that the protein hubs are an essential component of the PPI network to have deep insights into the disease infection mechanisms. The common and unique host hubs from the 3 MPXV strains have been discussed below.

**Figure 4:**
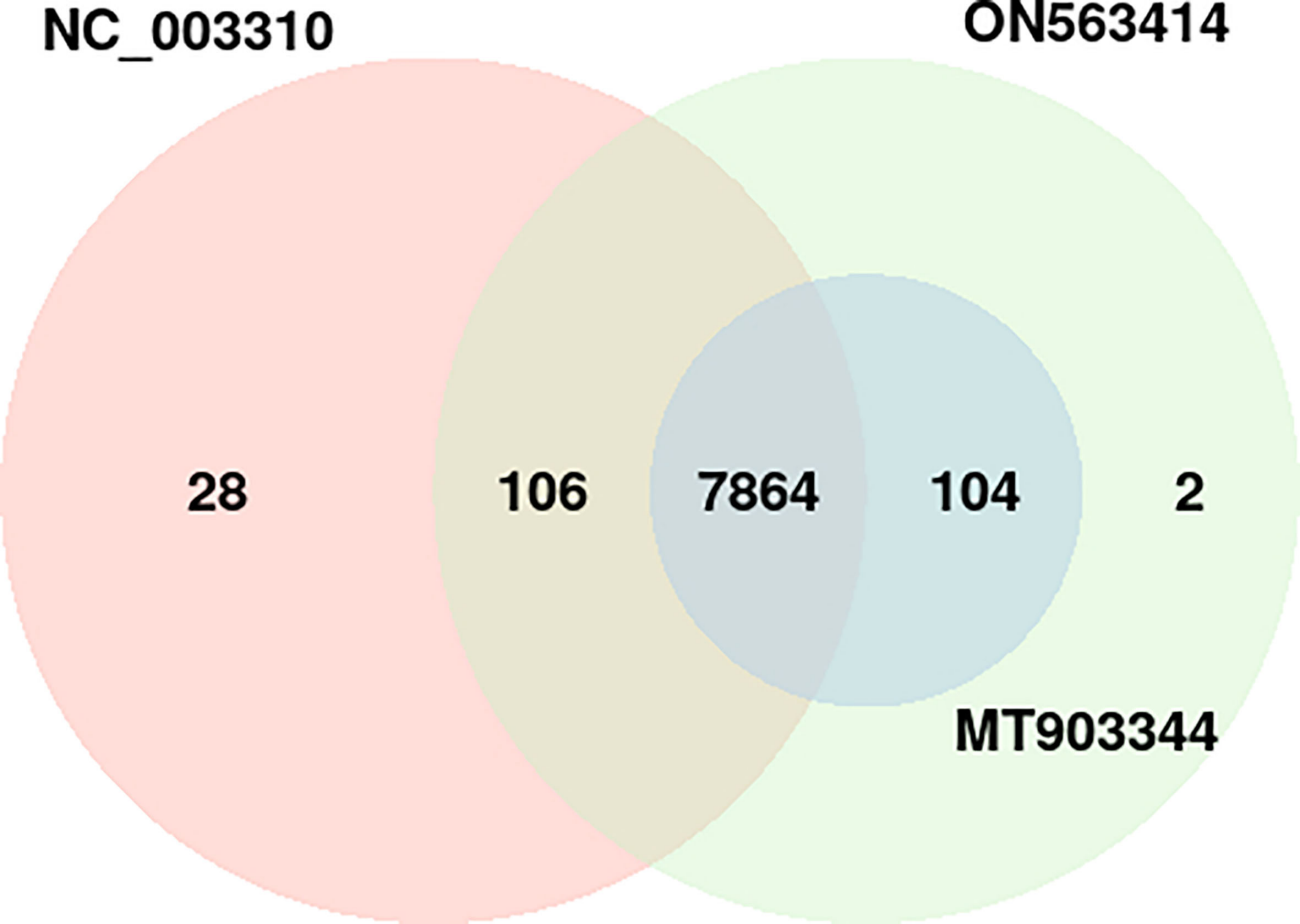
Distribution of the host protein hubs among the three strains of monkeypox virus (NC_003310, ON563414, and MT903344). *p*-value of the overlap region is 0.

In the common protein hubs analysis, we found 7,864 host proteins that were common in all the 3 strains (**Figure 4**). Of these host proteins, 7027 proteins served as hubs, with a degree ranging from 2 to 58. From the common host hubs, we analyzed top 20 common host hubs, with an average degree of 57 (**Supplementary Material 1**, **Sheet 2**). Most of the protein hubs (Q7Z2Y5, Q92918, Q9UKE5, Q8IVH8, O75962, and O95819) were found to belong to protein kinase family. The protein kinase R (PKR) is the major antiviral target for the poxviruses, and its expression is ubiquitous in host cells (29). Various studies demonstrated the role of PKR in antiviral immune responses (including inhibition of viral replication) and regulation of virus-induced apoptosis by suppressing protein synthesis and phosphorylation of eIF2α (eukaryotic initiation factor 2α) (30, 31). The protein hubs O14578, Q5VT25, Q6DT37, and Q9Y5S2 belonged to AGC-kinase C-terminal. AGC-kinases are the most diverse, evolutionary conserved groups in humans and serve as the potential targets for analyzing various human diseases including cancer, neural disorders, and viral infections (32)C-terminal domain, belonging to RSK (Ribosomal S6 Kinase) subfamily, regulates the activation of RSK kinases by the process of phosphorylation, which further phosphorylates the substrates that play a key role in cell differentiation, proliferation, and survival. RSK family proteins are also implicated in cellular signaling cascades such as mitogen-activated protein kinase (MAPK) (33). Other protein hubs such as P17252, P05129, and P05771 belonged to C2 domain, a calcium-dependent binding motif, which is involved in membrane trafficking, and signal transduction (34, 35). Various other host hubs (P42684, P41240, P06239, P06241, P09769, P42685, and P12931) belonged to Src-homology (SH) domains: SH3 and SH2. SH2 and SH3 domains regulate the kinase activity and are involved in tyrosine kinase signaling in the cell (36). Researchers in the past demonstrated that the interaction of SAP (adaptor-like molecule) SH2 and FynT (cytoplasmic Src-related protein tyrosine kinase) SH3 domains is crucial for the regulation of immune cell function (37). The immune role of these protein hubs against the viral infections exhibits high significance in the interactome study and better understanding the host defense mechanism. The significant individual hub proteins have been illustrated in **Supplementary Figures S3-S7**.

Apart from the common hubs, 104 protein hubs were found to be common between MT903344 and ON563414 strain, while 106 protein hubs were in consensus between NC_003310 and ON563414 strain.

In terms of unique host hubs, we found 28 hubs that were found only in NC_003310 strain, involved in 36 PPIs with 5 pathogen proteins (**Supplementary Figure S1**), while 2 hubs were found unique to ON563414 strain interacting with 2 pathogen proteins (**Supplementary Figure S2**). However, the functional annotation revealed no significant differences for these hubs in comparison to the common host hubs.

### 3.4 Gene ontology analysis unravels the involvement of human proteins in antiviral immune response

**Figure 5:**
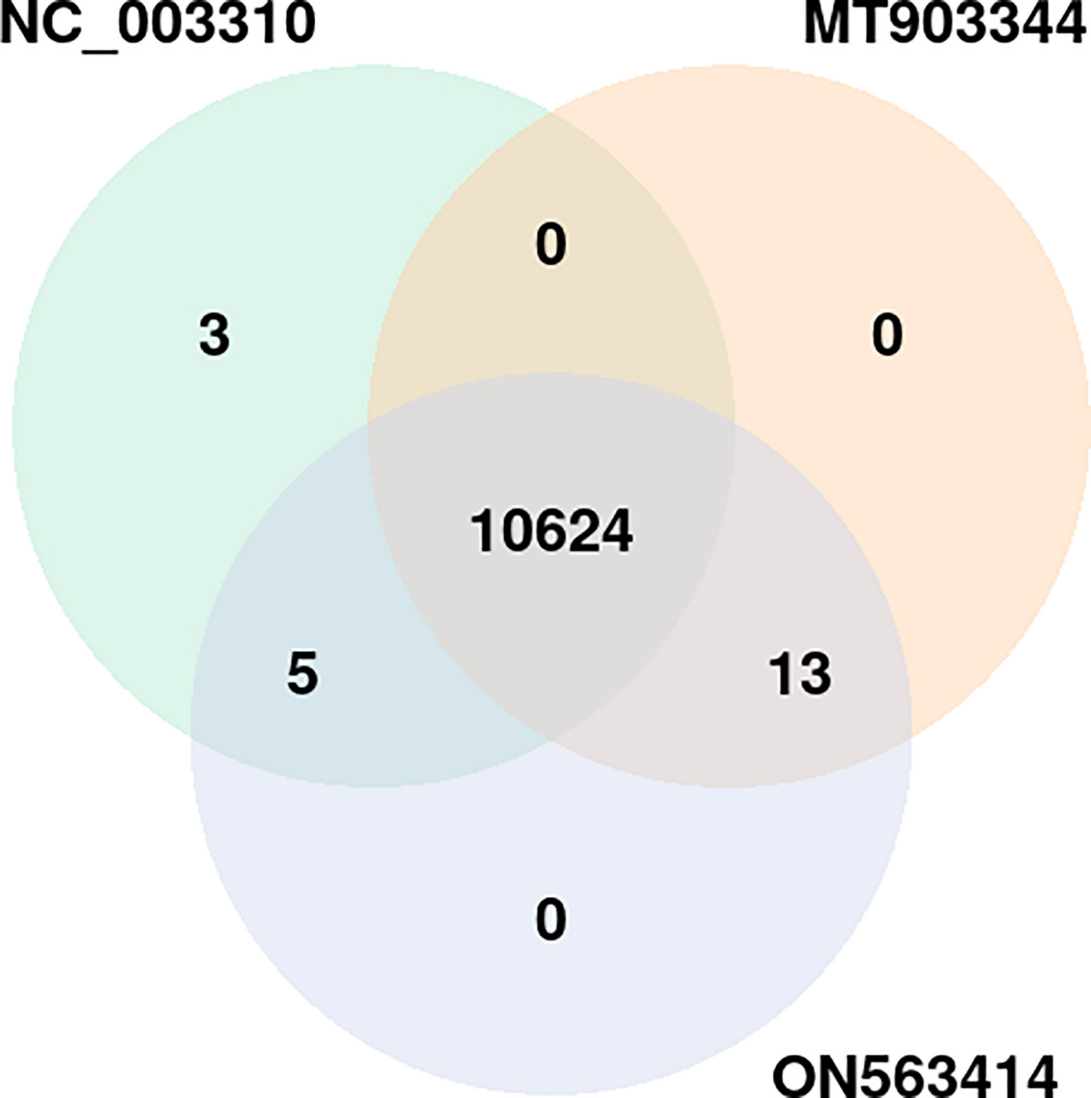
Distribution of common and unique gene ontology terms among the three strains of monkeypox virus (NC_003310, ON563414, and MT903344). *p*-value of the overlap region is 0.

**Figure 6:**
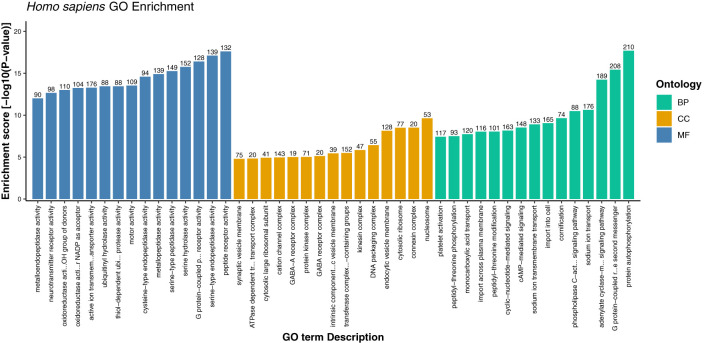
Gene ontology enrichment of host proteins representing top 15 terms from each category i.e., biological process, cellular component, and molecular function on the basis of enrichment score.

To get deeper insights into the immune defense mechanism, we performed the GO enrichment analysis of the host proteins involved in the interactions. The enrichment analysis revealed that a total of 10,645 GO terms including three major GO categories including, biological processes (BP), cellular component (CC), and molecular function (MF) were significantly enriched. For comparative analysis, the strain-wise distribution of these GO terms is depicted in **Figure 5**. Of these GO terms, 10,624 terms were commonly enriched among all the 3 strains (**Figure 6**). On the other hand, 13 GO terms were commonly represented between MT903344 and ON563414, 5 GO terms were commonly enriched between NC_003310 and MT903344, and 3 GO terms were uniquely enriched only in NC_003310 (**Supplementary Material 2**, **Sheet 1**). The uniquely expressed GO terms including, DNA double-strand break processing involved in repair *via* single-strand annealing (GO:0010792), double-strand break repair *via* single-strand annealing (GO:0045002), and regulation of MyD88-dependent toll-like receptor signaling pathway (GO:0034124) play a significant role in the host. Various studies suggest that ascorbic acid and MyD88-dependent toll-like receptor signaling plays an important role in innate antiviral immune control during viral infection. The toll-like receptors recognize specific sequences in uridine and guanosine rich single stranded RNA, thus facilitating the cascade of immune inflammatory reactions during viral infection (38–41).

**Figure 7:**
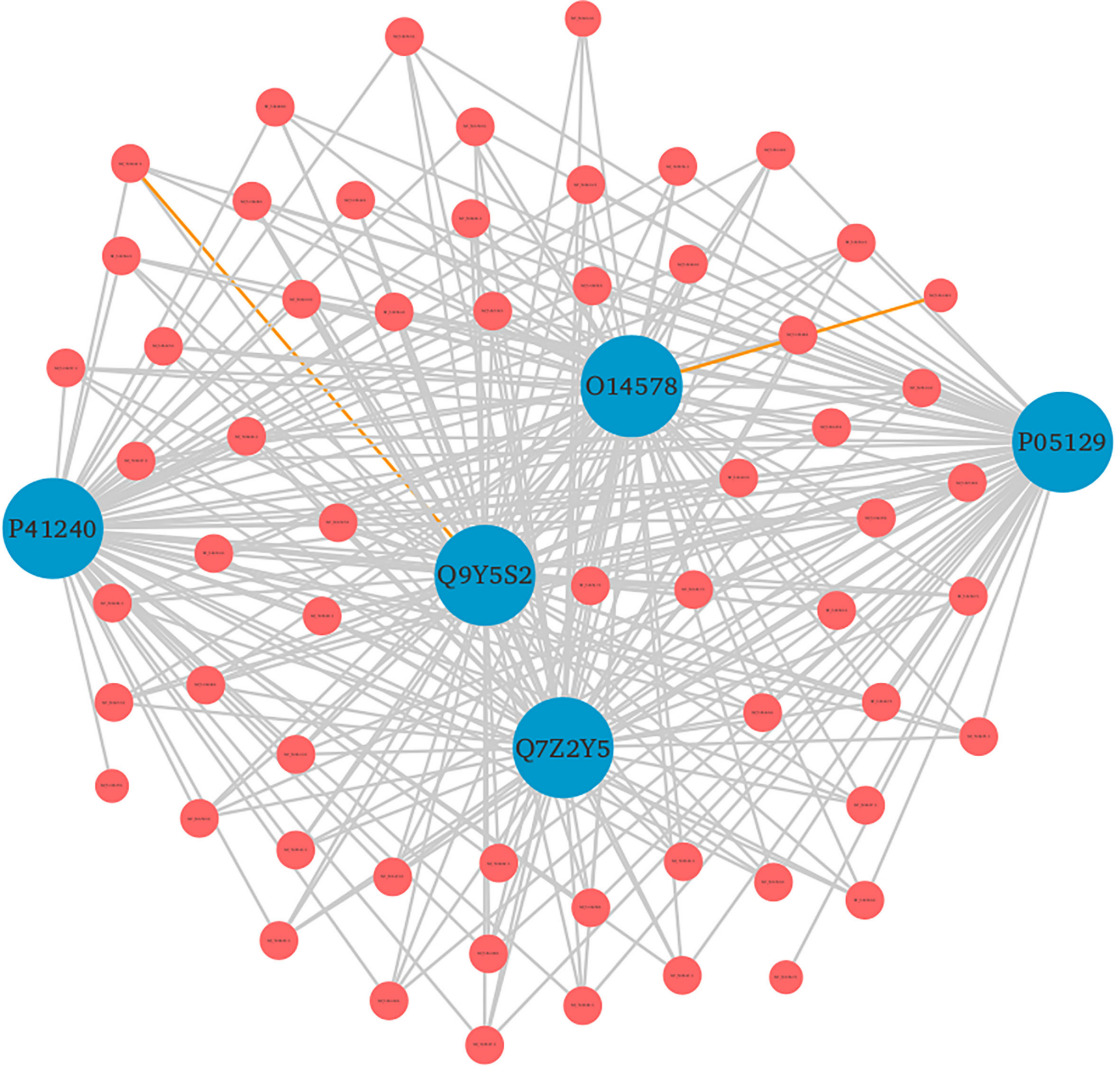
Top 5 human protein hubs based on their degree. The hub protein “P05129” was found to be critically involved in various immune responses. Blue nodes represent host proteins, and red nodes are pathogen proteins. Orange edges depict the interactions from interolog-based approach, while grey edges belong to domain-based approach.

Furthermore, the detailed analysis revealed that based on the enrichment score [-log10 (p-value)], ~76% of the significantly enriched GO terms represented the biological processes (BP) category. The highly enriched GO terms in this category included protein autophosphorylation (GO:0046777), adenylate cyclase-modulating G protein-coupled receptor signaling pathway (GO:0007188), sodium ion transport (GO:0006814), phospholipase C-activating G protein-coupled receptor signaling pathway (GO:0007200), and platelet activation (GO:0030168) (**Supplementary Material 2**, Sheet 2). When a cell is infected with a virus, various cellular signaling pathways including JAK/STAT, MAPK, ERK, and others utilize the protein autophosphorylation processes that lead to the production of several cytokines and chemokines (42–44). In agreement with other findings, we reported that protein phosphorylation is the most important first line of defense against viral infection and up to ~30% of all human proteins may undergo kinase action modification (42, 43, 45). Since both viral and cellular kinases are crucial for viral infection, thus these mechanisms along with exploration of signaling pathways including G-protein coupled and calcium-mediated signaling can be targeted for monkeypox related therapeutic drugs to treat the infected patients (46–48). Furthermore, platelets are one of the key players that regulate innate immune antiviral response. Studies suggest that the platelet activation might play an important role in viral infections, and the low count of platelets during infection signifies their involvement in innate immune response to viral infection (49, 50). In consistence with these results, our findings also reported the significant enrichment of GO term related to the platelet activation. The human protein hub “P05129” was found to be enriched in platelet activation, and localized in cytoplasm of the host (**Figure 7**). Our analysis revealed that this protein is also actively involved in calcium signaling pathway. Therefore, our results clearly indicate the role of kinases, signaling mechanisms, transmembrane transport, and platelet activation in humans in response to infection with different strains of MPXV.

In addition, under cellular component category, the host proteins were significantly enriched in 969 GO terms (~9%), and the most significant GO terms included nucleosome, connexin complex, cytosolic ribosome, endocytic vesicle membrane, and DNA packaging complex (**Supplementary Material 2**, Sheet 3). Under the molecular function category, 1594 GO terms (~15%) were overrepresented, including peptide receptor activity (GO:0001653), serine-type endopeptidase activity (GO:0004252), G protein-coupled peptide receptor activity (GO:0008528), serine hydrolase activity (GO:0017171), and serine-type peptidase activity (GO:0008236) (**Supplementary Material 2**, Sheet 4). The role of host proteases including serine type peptidase or endopeptidase activity has been well documented, especially in viral infections such as COVID-19 (51–54). Since the appearance of novel mutations demands accurate and precise drugs and vaccines, therefore target inhibition of several proteases specific GO terms reported in this study can be explored as a therapeutic approach against monkeypox virus infection. We anticipate that these findings might shed light onto the detailed understanding and exploration of various drug targets against monkeypox disease.

### 3.5 Crucial metabolic pathways in response to viral infection

**Figure 8:**
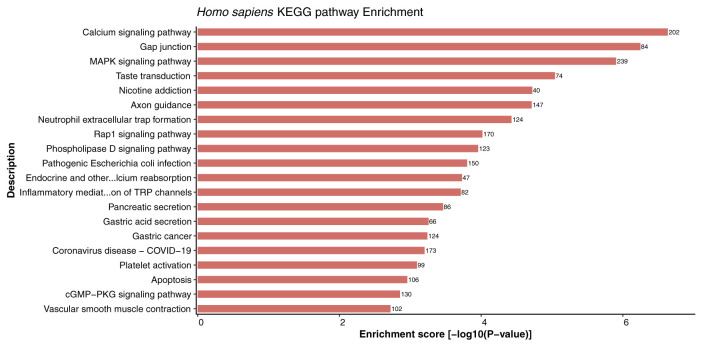
Depiction of significantly enriched pathways for human proteins involved in interactions with monkeypox virus proteins, based on enrichment score.

Kyoto Encyclopedia of Genes and Genomes (KEGG) enrichment analysis provides systematic and comprehensive understanding of gene functions, thus playing role in elucidation of various metabolic pathways associated with genomic functions in the organism. In this study, KEGG pathway enrichment analysis of the host proteins interacting with 3 different MPXV strains (NC_003310, MT903344, and ON563414) was performed. Remarkably, the host proteins were found to be enriched in 332 KEGG pathways in all the strains and no unique pathways were identified in any of the strains (**Supplementary Material 3**, Sheet 1). The highly enriched pathways included calcium signaling pathway (hsa04020), neuroactive ligand-receptor interaction (hsa04080), MAPK signaling pathway (hsa04010), gap junction (hsa04540), taste transduction (hsa04742), axon guidance (hsa04360), and nicotine addiction (hsa05033). The top 20 KEGG enriched pathways for host proteins are represented in **Figure 8**.

Cell signaling is an important component of a superior communication system that allows living cells to interact with one another and the extracellular (EC) environment. Calcium signaling pathways can trigger the genome-wide up-regulation of several calcineurin-dependent response element genes which further provides resistance to the pathogen (55). Interestingly, calcium signaling was reported to be the most enriched pathway in host proteins from 3 different strains. Furthermore, a study suggested that infection of rota-virus facilitates cell to cell signaling in the form of endogenous calcium waves that triggers the release of ADP by the virus-infected cells. The ADP release activates the receptors of neighbouring infected cells and leads to the generation of calcium signal, which activates the serotonin and chloride ion secretion, subsequently causing vomiting and diarrhoea. These reports indicate the critical role of calcium-mediated signaling in response to viral infection (56).

Furthermore, studies suggest that some host factors might not be necessary for the host, but they might be essential for virus replication, thus making them potential candidates for the generation of antiviral therapeutics/drugs. A number of viruses are known to activate the major cell signaling pathway— mitogen activated protein kinase (MAPK) pathway. It has been demonstrated that the MAPK interacting kinase 1 (MNK1) controls both cap-dependent and IRES-mediated mRNA translation, thus playing an important role in protein synthesis, and function in response to infection (57, 58). Additionally, we have reported the involvement of gap junction in host protein pathways, and evidence suggests that gap junctions allow both direct and indirect communication between cells in response to bacterial or virus infection. Formation of channels by these gap junction proteins play an essential role in many biological processes occurring during pathogen infection (59). Furthermore, the Toll-like receptors (TLRs) are membrane-bound and intracellular receptors that facilitate the identification of damage- and pathogen-associated molecular patterns (DAMPs and PAMPs). A study suggested that innate signaling pathways dependent on TLRs are activated by poxvirus infection (60). TLRs activated or targeted by poxvirus infection have both advantageous and adverse effects on the host. Utilizing TLR agonists and antagonists may help treat and prevent poxvirus infections. A study revealed that TLR7 agonists (imiquimod and resiquimod) enhance immune responses after smallpox vaccination (61). The metabolic pathway enrichment analysis also revealed that several host proteins are involved in majority of the interactions related to G-proteins and calcium-mediated signaling, MAPK mediated signaling, transport, inflammatory regulations, TLRs mediated regulation, hormonal signaling, and carbohydrate and fatty acid metabolism, thus indicating the role of various pathways in mediating interactions and defense responses of the host while interacting with the pathogen.

### 3.6 Cytoplasm and nucleus: Dynamic cellular compartments for host immune response

**Figure 9:**
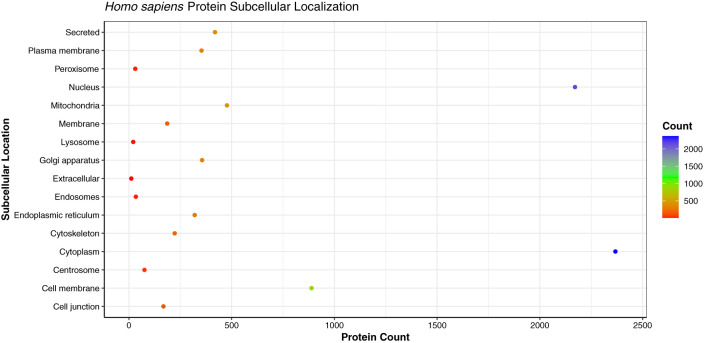
Dot plot representing the distribution of the subcellular localizations of human proteins involved in interactions with monkeypox virus.

Being the crucial components of cellular processes, the translocation of proteins is essential to maintain the cellular homeostasis and regulate the function of other molecules in vicinity (62). As the proteins perform various functions in different cellular compartments, the prediction of subcellular localization of proteins enhances the understanding of protein function in the specific cell organelle. We analyzed the subcellular localization of the host proteins obtained from the PPI predictions of three strains, which showed that the primary subcellular localization of 2,368 human proteins was cytoplasm (29.22%), followed by 2,171 proteins localized in nucleus (26.79%), and 889 proteins in cell membrane (10.97%) (**Figure 9**). The analysis revealed that the host proteins localized in cytoplasm were found to be interacting with 111 pathogen proteins, involved in 32,222 PPIs. On the other hand, the nuclear proteins were associated with 106 pathogen proteins, involved in 24,258 PPIs. 1,040 human proteins were found to be localized in multiple (more than 2) cellular locations, while 960 proteins had dual localizations i.e., localized in two different cellular compartments according to their function. The detailed subcellular localizations of human proteins can be found in **Supplementary Material 4**, **Sheet 1**.

The high number of human proteins localized in the cytoplasm and nucleus are in line with the previous studies for human immune response against viral infections. Researchers in the past have demonstrated the accumulation of MxA interferon proteins in cytoplasm against DNA/RNA viruses, while the MxB proteins were found to be localized in the nucleus and outer nuclear membrane, thus inhibiting the herpesvirus and human immunodeficiency virus (HIV) (63, 64). A study described the role of pattern recognition receptors (PRRs) in the cytosol during viral infection. In humans, RIG-I and AIM2 were identified as an effective PRRs against vaccinia virus, a Poxviridae family virus. The proteins associated with PRRs distinguish the viral genome in the cell cytoplasm and further activate the host signaling cascades (65).

### 3.7 Identification of candidate drugs/vaccines: Need of the hour

**Figure 10:**
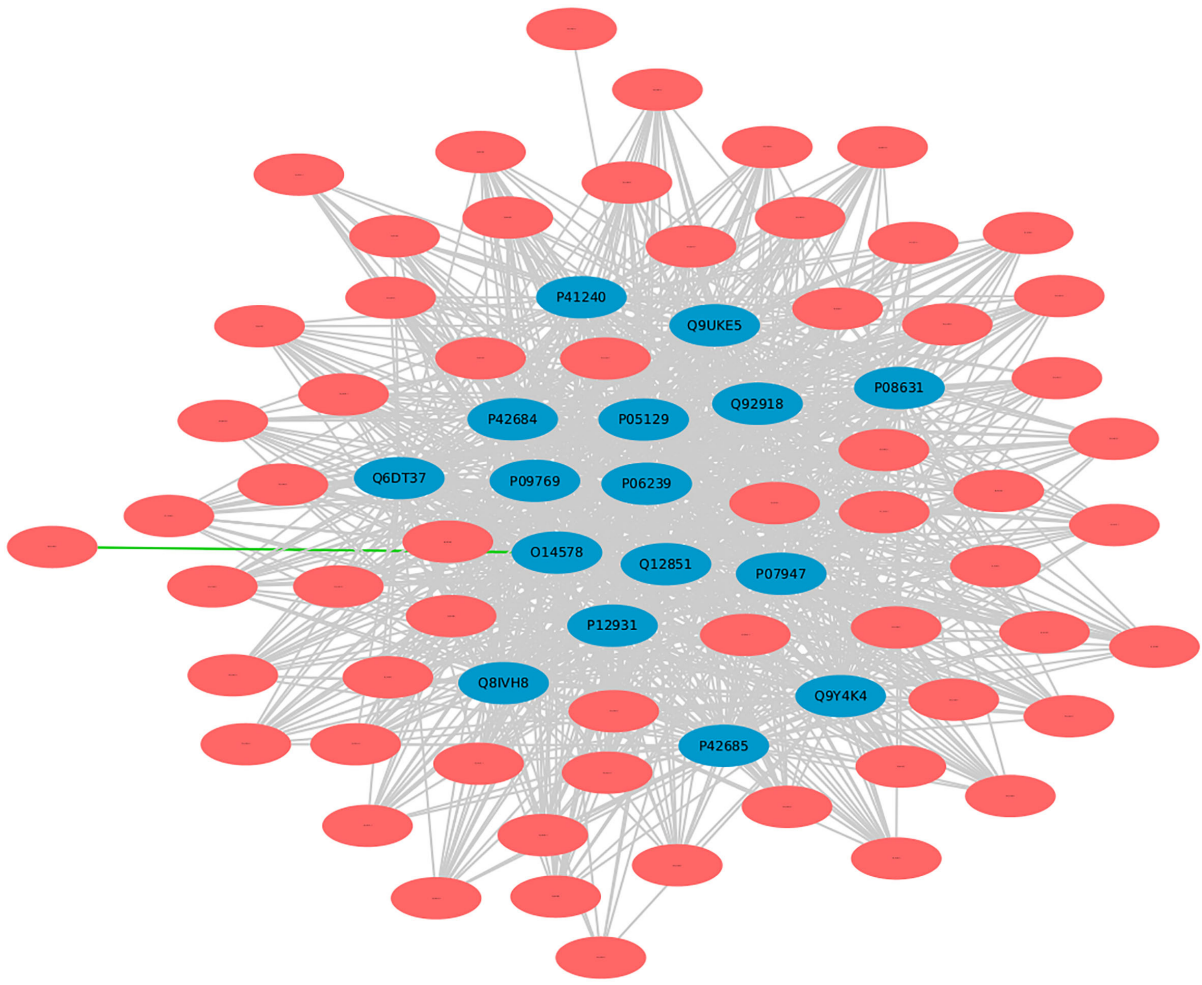
Human targets for the drug “Fostamatinib”. Blue nodes represent host proteins, and red nodes are pathogen proteins. Green edges depict the interactions from interolog-based approach, while grey edges belong to domain-based approach.

In DrugBank, a categorized human pathogen interactions group has been registered in drug entries (66). We analyzed the top 20 human protein hubs for the identification of the potential drug targets against MPXV. The analysis identified a drug target protein Q8IVH8, Mitogen-activated protein kinase kinase kinase 3 (MAP3K3), which is involved in cell signaling pathways and known to be activated by majority of the viruses has been linked with Fostamatinib drug (DB12010). Apart from this, 15 other human proteins including O14578, P05129, Q9UKE5, P41240, and others were identified as the potential targets of Fostamatinib (**Figure 10**). Reports say that these kinases play an important role in regulation of translational processes during the infection (58). Computational studies and docking analysis by various researchers suggest that Fostamatinib may be taken into consideration as one of the potential candidates to reduce the immunopathogenesis of COVID-19. Fostamatinib has also been reported to inhibit neutrophils extracellular traps induced by the plasma of COVID-19 patient (67–69).

Furthermore, another mined drug target protein “P05129”, Protein kinase C gamma type, is linked to both Tamoxifen (DB00675) and Fostamatinib (DB12010). Protein kinase C is a serine/threonine protein kinase involved in cell signal transduction process, thus play an important role in viral entry (70). Studies suggest that Tamoxifen (TAM), an estrogen receptor modulator, is involved in a diverse range of biological processes and has already been reported as a promising candidate for repurposing against zika virus and hepatitis C virus, a flavivirus, and COVID-19 as it provides complete protection against severe acute respiratory syndrome infections (71, 72). The protein “P05129” was also found to be localized in the cytoplasm and involved in platelet activation and calcium signaling, thus being a potential candidate for further molecular interaction experiments. Several other drugs including Bryostatin 1 (DB11752), Aprinocarsen (DB06451), and Perifosine (DB06641) were also reported to be involved in interactions with “P17252” protein (Protein kinase C alpha type). Studies suggest that these drugs are involved in decreasing viral infections including HIV-1 infection and viral production in human primary macrophages (73–76).

Additionally, the analysis of various drugs (Aprinocarsen, Bosutinib, Bryostatin 1, Dasatinib, Fostamatinib, Tamoxifen, and Zanubrutinib) revealed that these drugs belong to protein kinase family (protein kinase C alpha type, proto-oncogene tyrosine-protein kinase Src, and tyrosine-protein kinase BTK) that are involved in significant biological processes such as zinc ion binding, Sh3/sh2 adaptor activity, and protein tyrosine kinase activity. We also identified a few domains including C2 domain, AGC-kinase-C-terminal domain, SH2, SH3, and CAV1-binding motif that were common amongst these drugs. Various drug candidates predicted from our study are described in **Supplementary Material 4**, Sheet 2. We anticipate that the aforementioned drug target proteins predicted from our study could be considered as potential therapeutic candidates for finding treatments against MPXV infection.

## Data availability statement

The datasets presented in this study can be found in online repositories. The names of the repository/repositories and accession number(s) can be found in the article/**Supplementary Material**.

## Author contributions

RKau formulated and designed the research. RKat analyzed the data, developed prediction models, and performed functional analysis of the data, validations, literature mining etc. Writing - original draft preparation, RKat and SK. writing - review and editing, RKau. visualization, RKat, SK and RKau. supervision, RKau. project administration, RKau. funding acquisition, RKau. All the authors have read and agreed to the published version of the manuscript. All authors contributed to the article and approved the submitted version.

## Supplementary material

Supplementary Figure 1 Protein interaction network representing the unique host hubs for NC_003310 strain. Blue nodes are host proteins, and red nodes are pathogen proteins. Grey edges depict the interactions from domain-based approach.

Supplementary Figure 2 Visualization of unique host hubs for ON563414 strain. Blue nodes represent host proteins, and red nodes are pathogen proteins. Grey edges depict the interactions from domain-based approach.

Supplementary Figure 3–7 Interaction networks of host hub protein with MPXV proteins. Blue nodes represent host proteins, and red nodes represent pathogen proteins. Grey and orange edges depict domain- and interolog-based interactions, respectively.
