# Supplementary figures and images for: Deciphering the complete human-monkeypox virus interactome: Identifying immune responses and potential drug targets
Source: Front Immunol. 2023 Mar 27;14:1116988. doi: 10.3389/fimmu.2023.1116988 (PMC10083500; doi:10.3389/fimmu.2023.1116988)

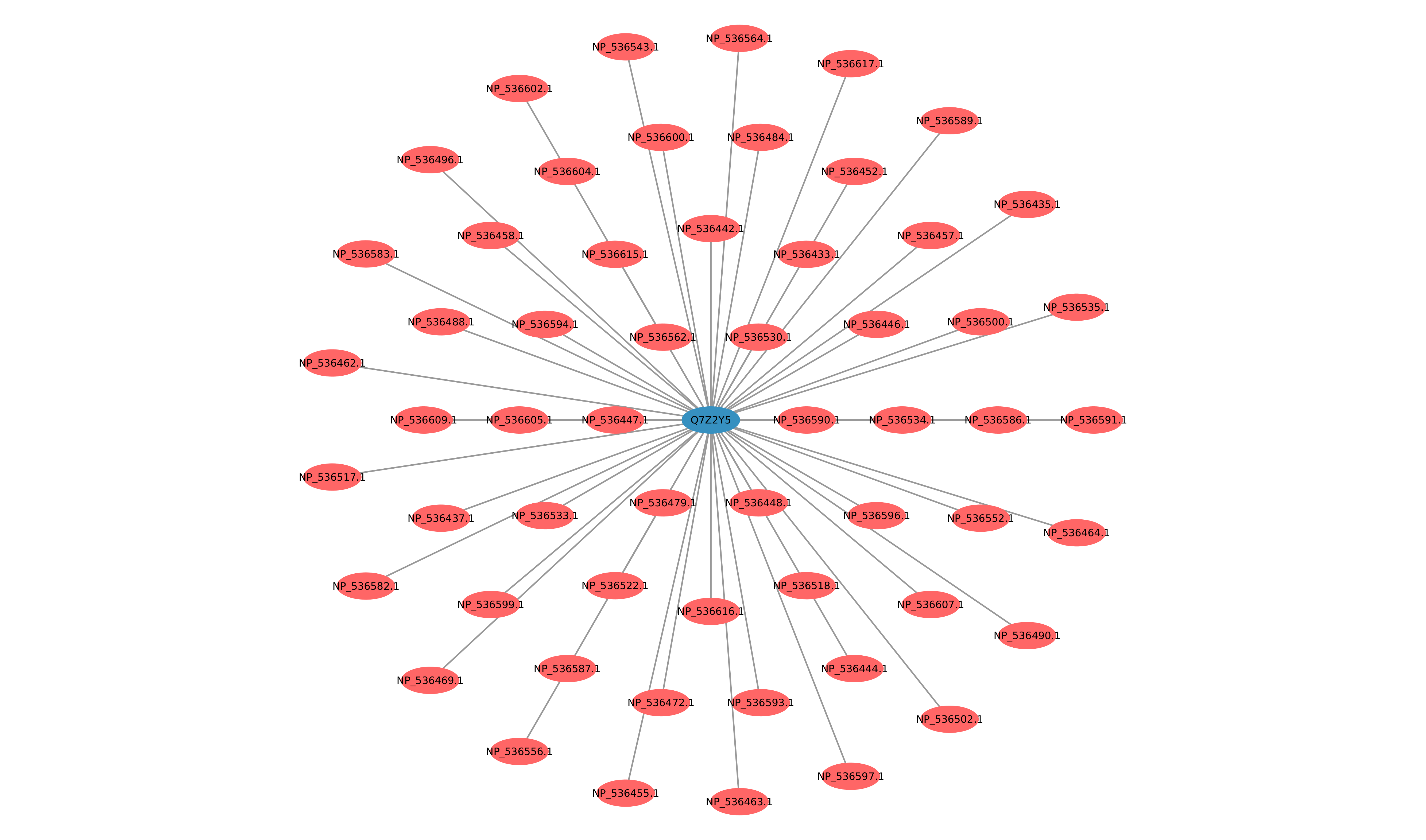

Supplement: Supplementary Figure 1 — Protein interaction network representing the unique host hubs for NC_003310 strain. Blue nodes are host proteins, and red nodes are pathogen proteins. Grey edges depict the interactions from domain-based approach. [file DataSheet_1.zip › Supplementary Data-Figures/Supplementary Figure S6.png]

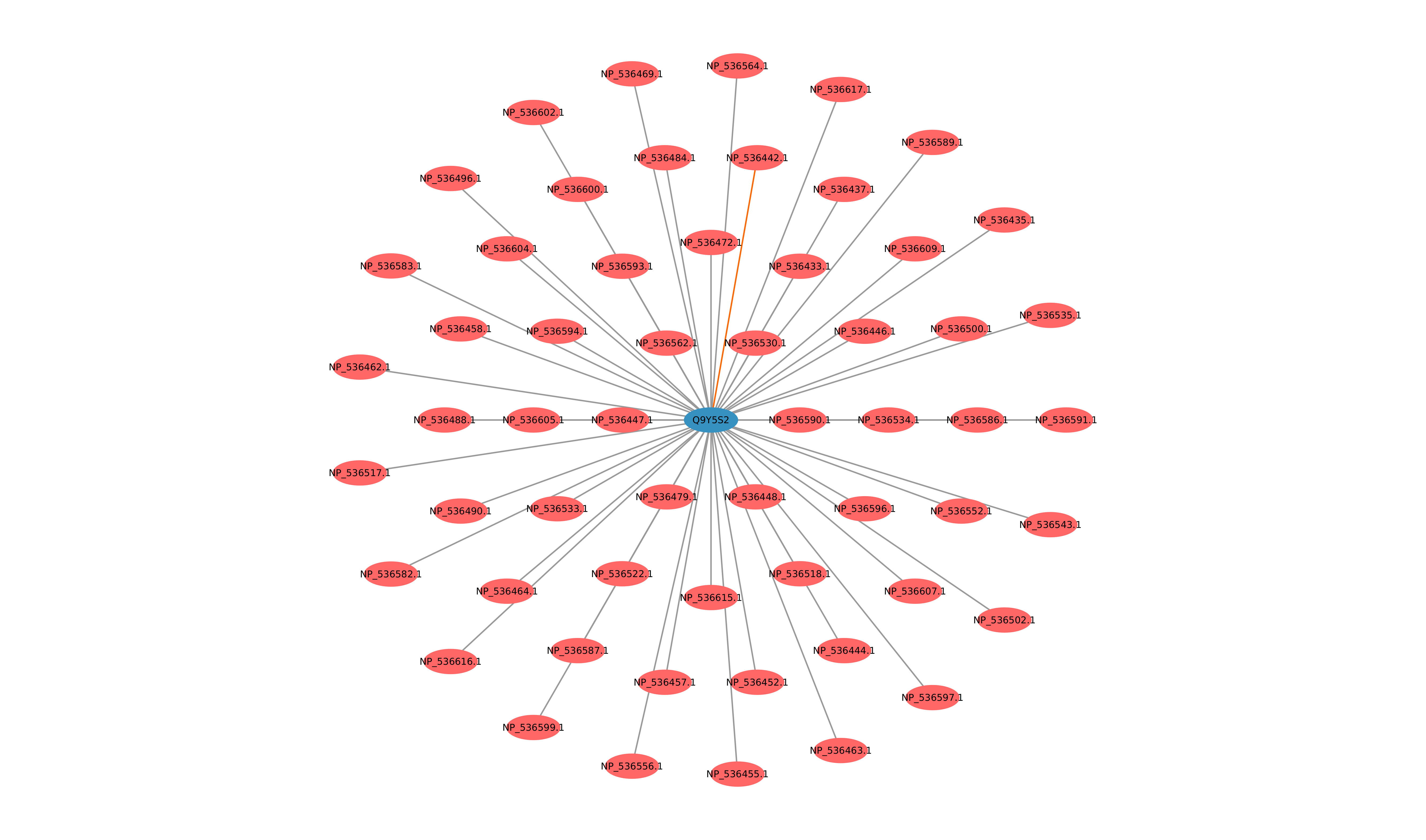

Supplement: Supplementary Figure 1 — Protein interaction network representing the unique host hubs for NC_003310 strain. Blue nodes are host proteins, and red nodes are pathogen proteins. Grey edges depict the interactions from domain-based approach. [file DataSheet_1.zip › Supplementary Data-Figures/Supplementary Figure S7.png]

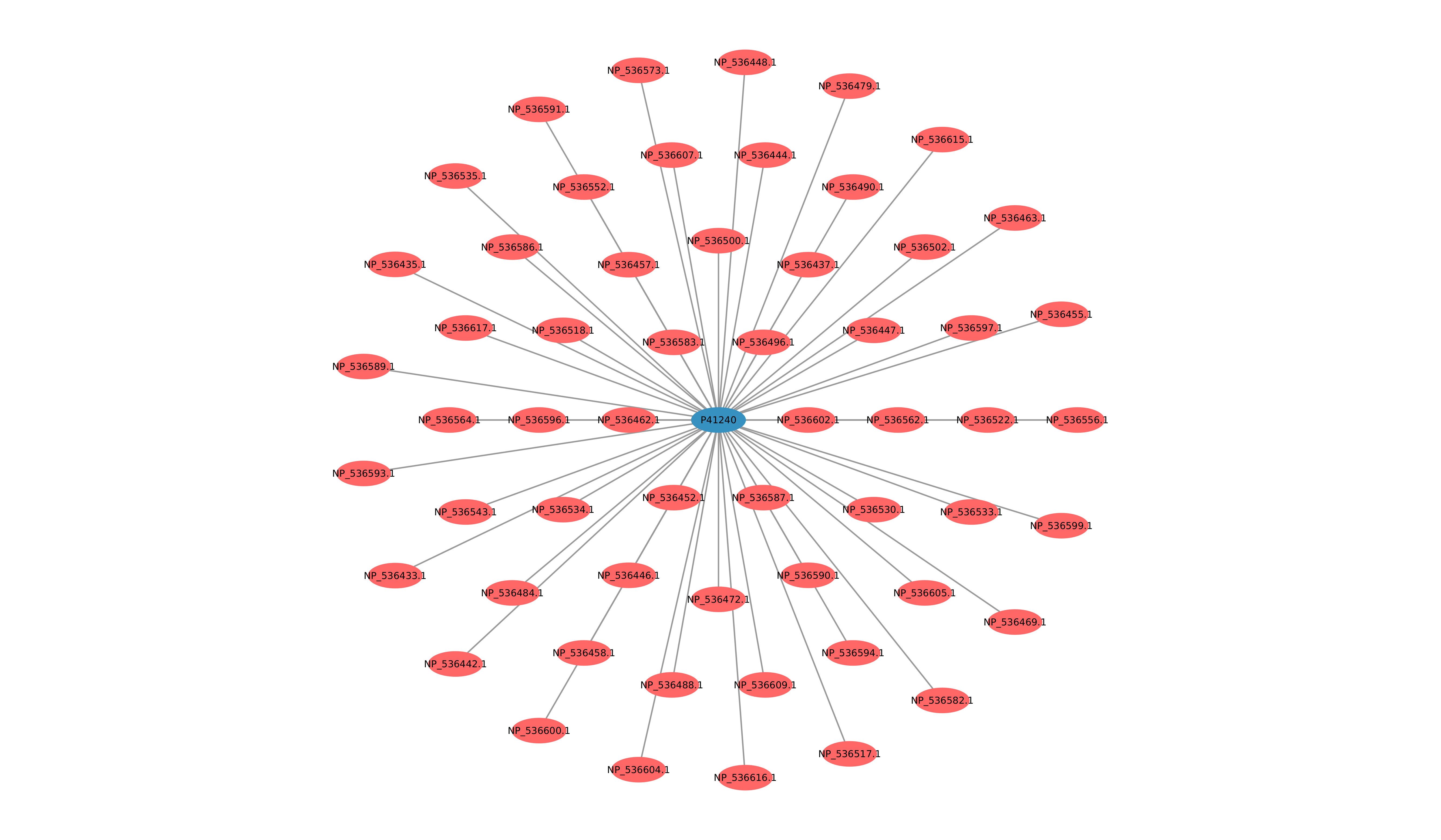

Supplement: Supplementary Figure 1 — Protein interaction network representing the unique host hubs for NC_003310 strain. Blue nodes are host proteins, and red nodes are pathogen proteins. Grey edges depict the interactions from domain-based approach. [file DataSheet_1.zip › Supplementary Data-Figures/Supplementary Figure S5.png]

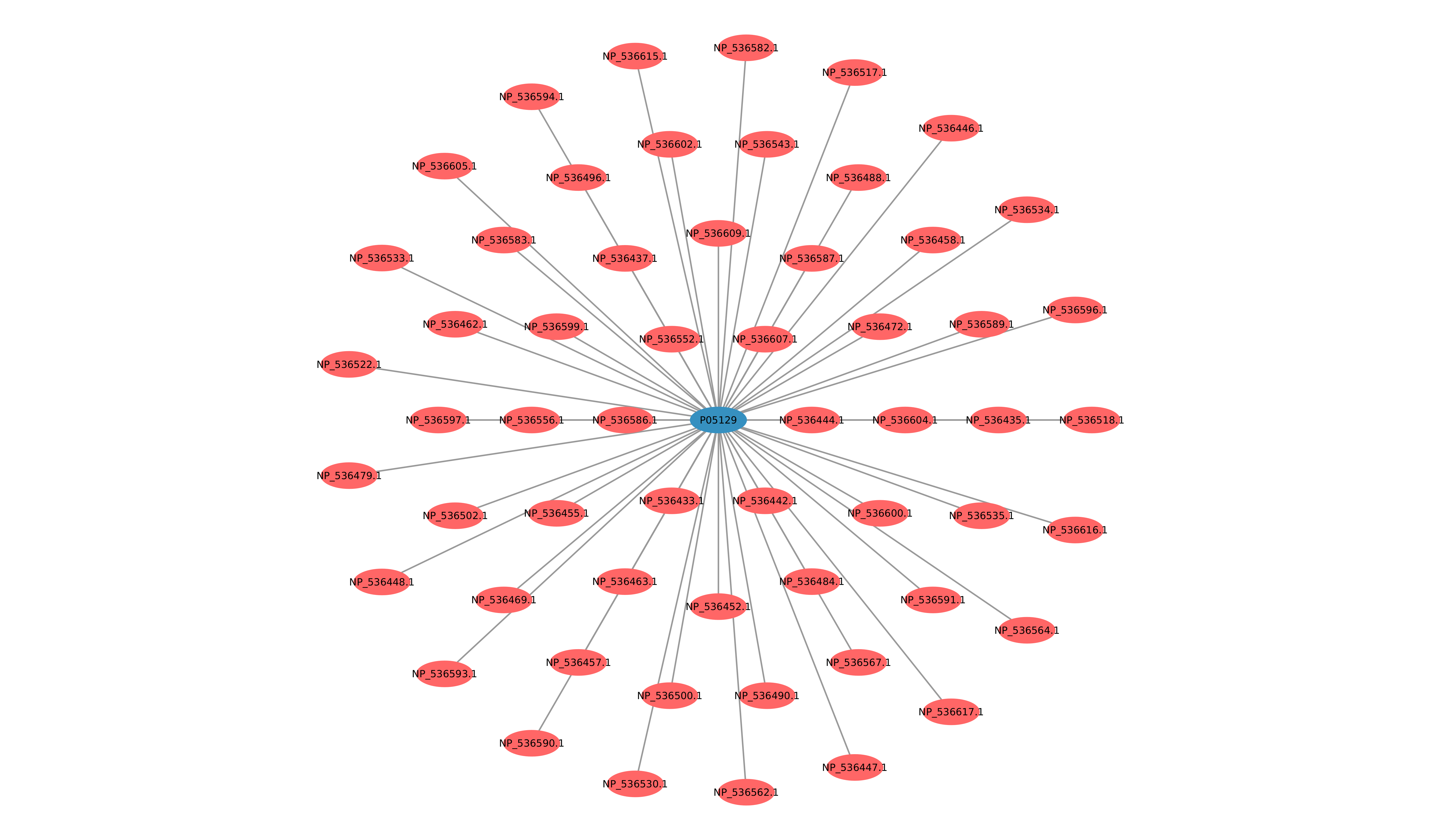

Supplement: Supplementary Figure 1 — Protein interaction network representing the unique host hubs for NC_003310 strain. Blue nodes are host proteins, and red nodes are pathogen proteins. Grey edges depict the interactions from domain-based approach. [file DataSheet_1.zip › Supplementary Data-Figures/Supplementary Figure S4.png]

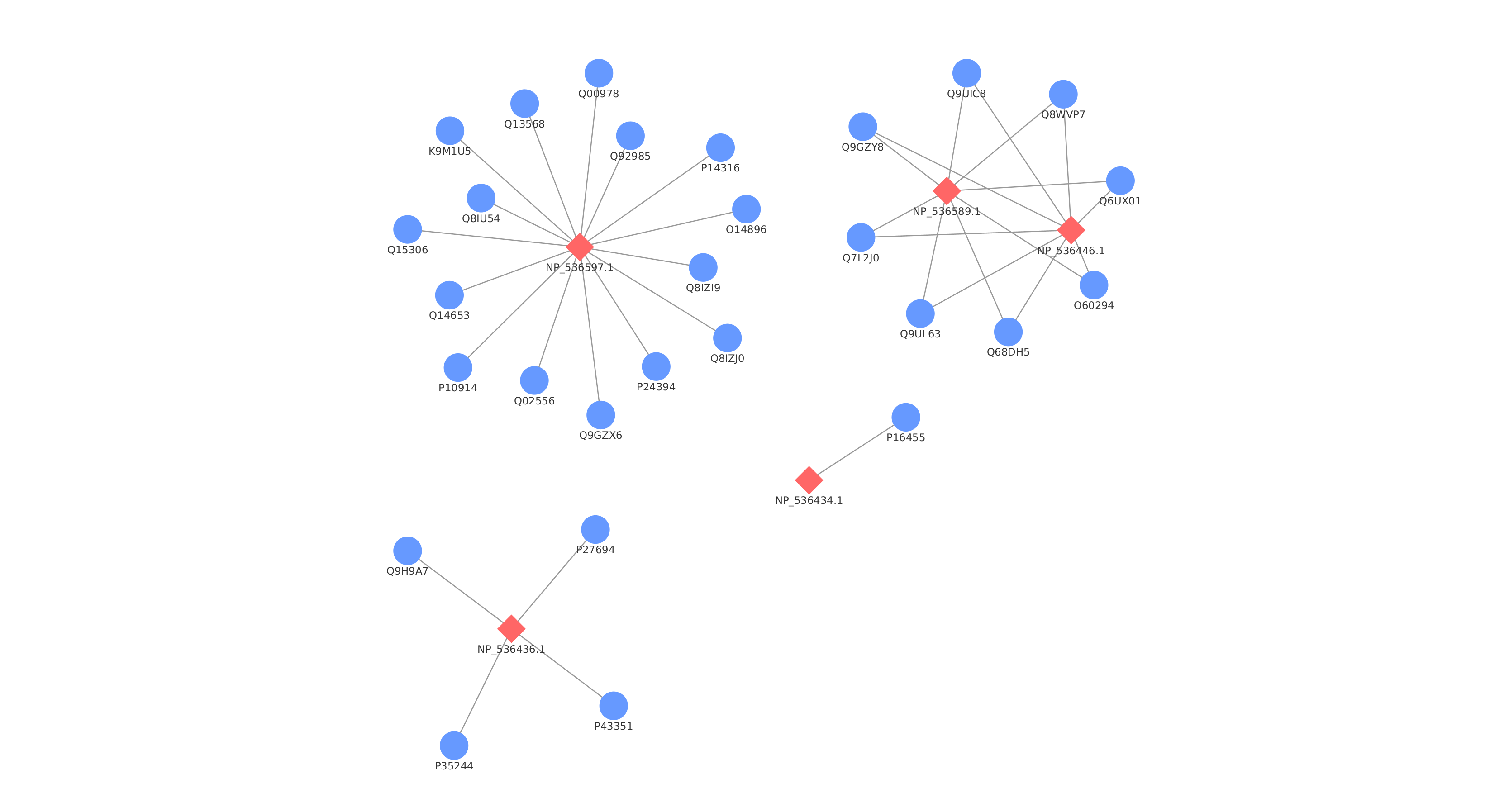

Supplement: Supplementary Figure 1 — Protein interaction network representing the unique host hubs for NC_003310 strain. Blue nodes are host proteins, and red nodes are pathogen proteins. Grey edges depict the interactions from domain-based approach. [file DataSheet_1.zip › Supplementary Data-Figures/Supplementary Figure S1.png]

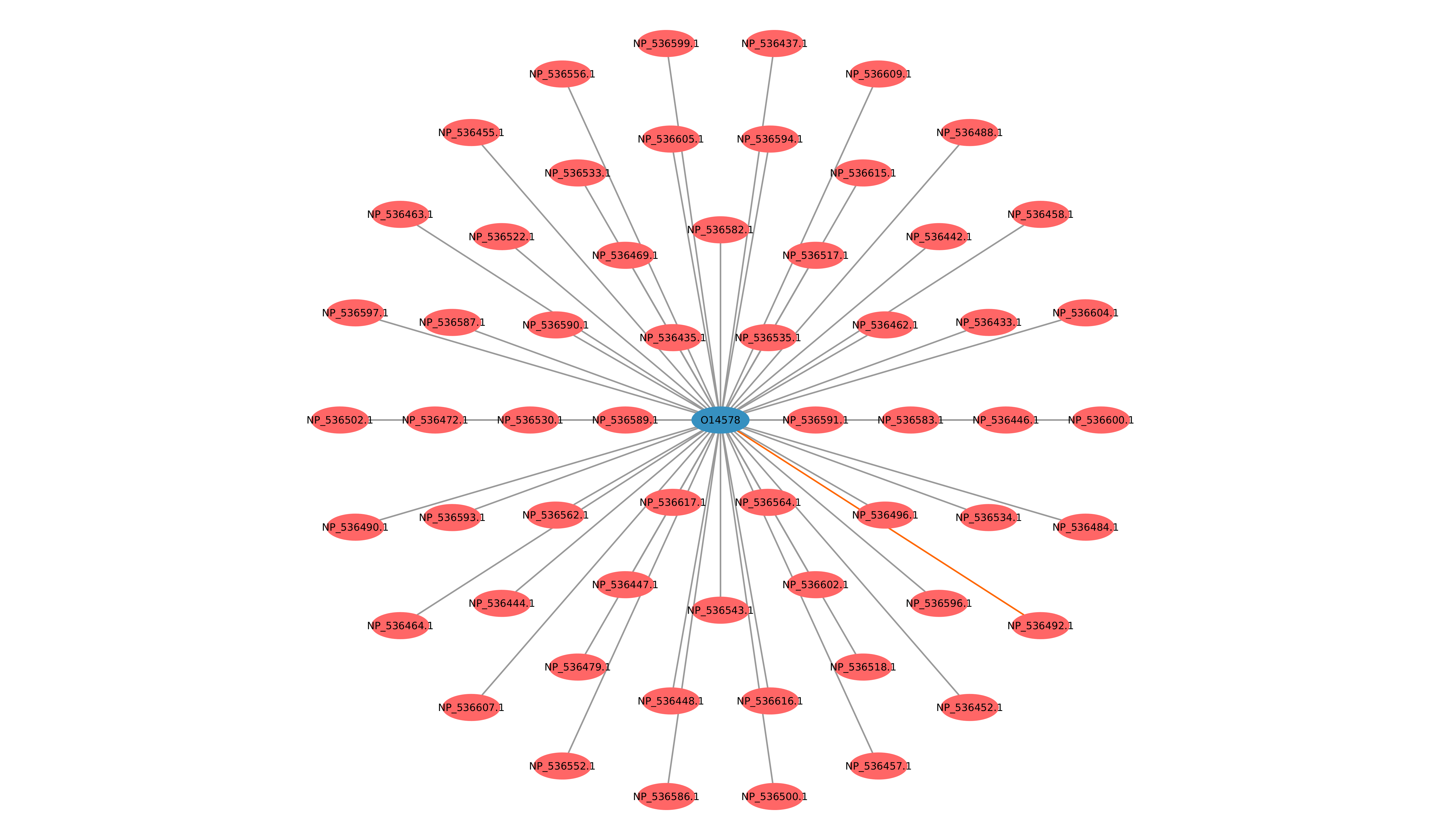

Supplement: Supplementary Figure 1 — Protein interaction network representing the unique host hubs for NC_003310 strain. Blue nodes are host proteins, and red nodes are pathogen proteins. Grey edges depict the interactions from domain-based approach. [file DataSheet_1.zip › Supplementary Data-Figures/Supplementary Figure S3.png]

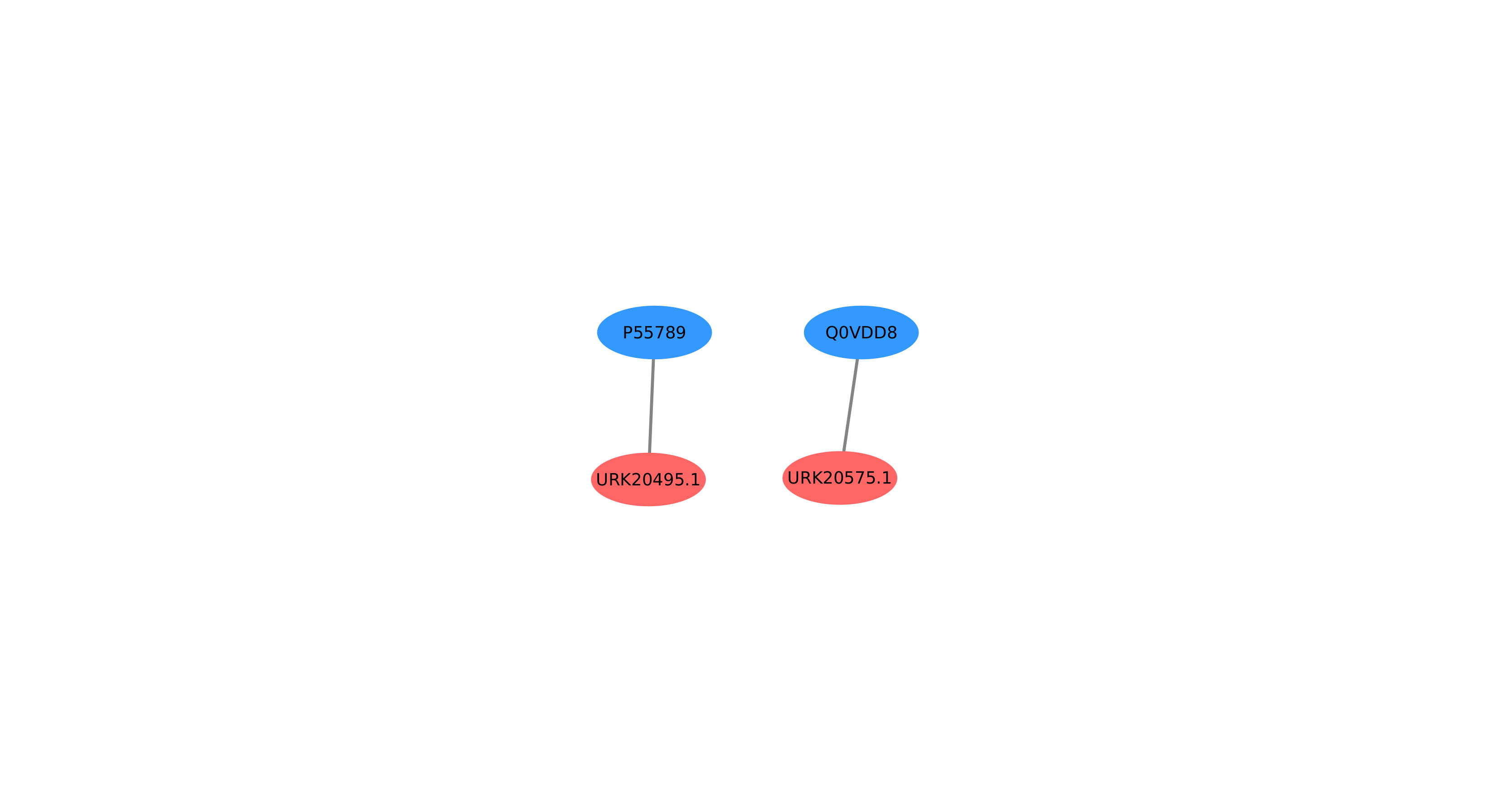

Supplement: Supplementary Figure 1 — Protein interaction network representing the unique host hubs for NC_003310 strain. Blue nodes are host proteins, and red nodes are pathogen proteins. Grey edges depict the interactions from domain-based approach. [file DataSheet_1.zip › Supplementary Data-Figures/Supplementary Figure S2.png]
